# Exonic mutations in cell–cell adhesion may contribute to CADASIL-related CSVD pathology

**DOI:** 10.1007/s00439-023-02584-8

**Published:** 2023-07-08

**Authors:** Paul J. Dunn, Rodney A. Lea, Neven Maksemous, Robert A. Smith, Heidi G. Sutherland, Larisa M. Haupt, Lyn R. Griffiths

**Affiliations:** 1 Genomics Research Centre, Centre for Genomics and Personalised Health, School of Biomedical Sciences, Faculty of Health, Queensland University of Technology (QUT), 60 Musk Ave, Kelvin Grove, QLD 4059 Australia; 2 Faculty of Health Sciences and Medicine, Bond University, 15 University Drive, Robina, Gold Coast, QLD 4226 Australia; 3 ARC Training Centre for Cell and Tissue Engineering Technologies, Queensland University of Technology (QUT), Brisbane, Australia; 4 Max Planck Queensland Centre for the Materials Sciences of Extracellular Matrices, Brisbane, Australia

## Abstract

Cerebral autosomal dominant arteriopathy with subcortical infarcts and leukoencephalopathy (CADASIL) is a condition caused by mutations in *NOTCH3* and results in a phenotype characterised by recurrent strokes, vascular dementia and migraines. Whilst a genetic basis for the disease is known, the molecular mechanisms underpinning the pathology of CADASIL are still yet to be determined. Studies conducted at the Genomics Research Centre (GRC) have also identified that only 15–23% of individuals clinically suspected of CADASIL have mutations in *NOTCH3.* Based on this, whole exome sequencing was used to identify novel genetic variants for CADASIL-like cerebral small-vessel disease (CSVD). Analysis of functionally important variants in 50 individuals was investigated using overrepresentation tests in Gene ontology software to identify biological processes that are potentially affected in this group of patients. Further investigation of the genes in these processes was completed using the TRAPD software to identify if there is an increased number (burden) of mutations that are associated with CADASIL-like pathology. Results from this study identified that cell–cell adhesion genes were positively overrepresented in the PANTHER GO-slim database. TRAPD burden testing identified *n* = 15 genes that had a higher number of rare (MAF < 0.001) and predicted functionally relevant (SIFT < 0.05, PolyPhen > 0.8) mutations compared to the gnomAD v2.1.1 exome control dataset. Furthermore, these results identified *ARVCF*,* GPR17*,* PTPRS*, and *CELSR1* as novel candidate genes in CADASIL-related pathology. This study identified a novel process that may be playing a role in the vascular damage related to CADASIL-related CSVD and implicated *n* = 15 genes in playing a role in the disease.

## Introduction

CADASIL is the most common monogenic cause of stroke and vascular dementia in the world and was originally estimated to have a prevalence of ~ 2–4 per 100,000. However, more recent work completed in the Exome Aggregation Consortium (ExAC) and Genome Aggregation Database (gnomAD) browsers has theorised that the prevalence could be up to 100-fold higher (~ 1:300) (Razvi et al., 2005; Rutten et al., 2016; Rutten et al., 2019). Clinical manifestations of CADASIL can be heterogeneous and include subcortical ischaemic events (60–80%); cognitive impairment including dementia and apathy (40–60%); migraine with/without aura (20–40%); mood disturbances including severe depression and manic episodes (~ 20%); motor disturbances, such as gait disturbances (90%), urinary incontinence (80–90%) and pseudobulbar palsy (50%); and other neurological manifestations such as epilepsy/seizures (~ 10%) (Choudhary et al., 2013; Di Donato et al., 2017). Despite the heterogeneous nature of the phenotype, a definitive diagnosis of CADASIL is completed through either the identification of characteristic cysteine altering mutations between exons 2 and 24 in *NOTCH3*, or through the presence of granular osmiophilic material (GOM) identified by a tissue biopsy (Mancuso et al., 2020; Mizuta et al., 2017; Ueda et al., 2009).

Despite what is known with CADASIL, the molecular mechanisms causing disease are still poorly understood and it may be that there are novel genetic causes of a CADASIL or related CSVD pathology. The Genomics Research Centre (GRC) has been conducting diagnostic genetic testing for CADASIL since the late 1990s. In this time, only between 16 and 23% of CADASIL referred patients have a detected disease-causing mutation in *NOTCH3* (Maksemous et al. 2016; Dunn et al. 2020). This has led to alternative investigations to identify other potential genetic causes of CADASIL and disorders with related symptoms. Some examples focussed on investigating other genes responsible for monogenic CSVD, mitochondrial dysfunction and genes in the related Alzheimer’s disease pathways in clinically suspected CADASIL patients that had no *NOTCH3* mutation (Dunn et al. 2022a, 2022b). Whilst these investigations identified some genetic causes for the CADASIL-like clinical phenotype, there was still a large proportion of individuals which had no clear cause of disease.

To address this, we sought to investigate if there is an increased genetic variant load in specific biological processes within the *NOTCH3* negative CADASIL population. We hypothesised that there may be specific damaging genetic variants within biological processes which may be contributing to the CSVD phenotype (Lee et al. 2014).

## Methods

### Case cohort generation

Blood samples were chosen from CADASIL referred patients (*n* = 50) with no pathogenic *NOTCH3* mutations. All patients had approved diagnostic testing for CADASIL with their doctors and ethical approval for this study was obtained through the QUT HREC along with appropriate consents for the patient cohort (Approval Number 1800000611). Diagnostic testing was performed using the Genomics Research Centre (GRC) custom 5-gene panel where only the notch receptor 3 (*NOTCH3*) gene was analysed. Further screening of calcium voltage-gated channel subunit alpha 1A (*CACNA1A)*, ATPase Na^+^/K^+^ Transporting subunit Alpha 2 (*ATP1A2*), sodium voltage-gated channel alpha subunit 1 (*SCN1A*), and Potassium two pore domain channel subfamily K member 18 (*KCNK18*) (Maksemous et al. 2016), which have all previously been associated with other diseases, such as familial hemiplegic migraine and migraine with/without aura, episodic ataxia type 2, spinocerebellar ataxia type 6 and epilepsy, was also performed to rule out any conditions with overlapping symptoms to CADASIL. Whole exome sequencing was performed on each sample using the Ion AmpliSeq Exome RDY-kits (Carlsbad, Ca., USA) for library preparation, according to manufactures’ instructions (MAN0010084). Template preparation, enrichment, and chip loading were performed using the Ion P1 Hi-Q Chef Kit (Cat. Number A30011) and 540 Chips on the Thermo Fisher Scientific Ion Chef (Carlsbad, Ca., USA) targeted at 200 bp lengths. Sequencing was performed using the Ion Proton and Ion S5 + platforms with sequencing alignment (Hg19) and variant calling completed via the Ion Torrent software (Carlsbad, Ca., USA).

### Control cohort generation

The gnomAD v2.1.1 (*n* = 125,748) whole exome sequencing population was used in this study to match the case generate population. This included alignment to Hg19 to match the CSVD population. The gnomAD WES dataset comprised 125,748 unrelated individuals that were sequenced as part of various disease-specific and population genetic studies (Karczewski et al. 2020).

### Analysis—overrepresentation test

Initial analysis of the CSVD case dataset was based on extracting functionally affected variants using the vcfDART pipeline focussing on single-nucleotide variants (SNVs) that had a SIFT, PolyPhen2, and MutationTaster scores marked as “D” for deleterious and a MAF < 0.001 based on population databases, such as 1000 Genomes, ExAC, and GnomAD (Benton et al. 2019). Further annotation of these variants utilised the PredictSNP2 software where only variants with < 2 in silico pathogenicity prediction tools had a benign/tolerated classification. For insertion/deletion (indel) variants, these were kept if the MAF < 0.001.

To remove artefactual findings, from both the SNVs and the indels in the sequencing data, variants were also investigated based on an allele ratio (AR) calculation based on the allele count data and the following calculation:

$$AR=\frac{AC}{CD}$$

where AR is the allele ratio, AC is the count of the alternate allele count at one position and CD is the total coverage for that position. Heterozygous variants were only kept if the AR was ≥ 0.35 and ≤ 0.65 and homozygous mutations were ≥ 0.9. Furthermore, variants were filtered based on how often the same variant came up in different individuals where a conservative estimate of only variants in < 10% of the CSVD population (*n* ≤ 5 unrelated individuals) were deemed to be not sequencing error. The remaining mutations were kept for each sample and combined into a master list where information pertaining to the genes where each variant was included. Duplicate genes were removed and a reduced list of individual genes were then analysed using gene ontology software (http://geneontology.org/) that utilised the Protein Analysis Through Evolutionary Relationships (PANTHER) classification system to identify overrepresented pathways and processes (Thomas et al. 2003; Mi et al. 2013, 2016). Over-represented pathways were determined using Fisher’s exact test and the Benjamin–Hochberg procedure was used for false discovery rate (FDR). Only pathways with an FDR p value of < 0.05 were considered significant biological pathways and processes. For the gene ontology software used, only the top hits within the PANTHER GO-Slim Biological Processes were used. However, results for the PANTHER GO-Slim Molecular Function and PANTHER GO-Slim Protein Class also helped to correlate these findings.

### Analysis—TRAPD burden test

The top hits identified to have the most biological significance were then used and tested using TRAPD Burden testing software (Guo et al. 2018). TRAPD utilises the gnomAD population dataset and the rare variants obtained using WES or whole genome sequencing (WGS) (https://github.com/mhguo1/TRAPD). But in brief, TRAPD requires the WES data to be pre-processed using bcftools and mpileup to separate out (multinucleotide variants) MNVs and left-align the variants obtained from the Ion Torrent sequencing data. This was completed individually for each sample vcf which was then merged using vcf-merge and annotated using variant effect predictor (VEP) from ensembl. VEP also has a filter function which was used on the merged vcf file to only include variants from the genes in the pathways/processes of the top hits obtained from the burden test. Running TRAPD focussed only on SNVs, coverage depth (CD) > 10, SIFT ≤ 0.05, PolyPhen ≥ 0.8, and a MAF < 0.01 to filter variants based on being rare and functionally affecting the proteins. A SNP file was generated from the case cohort. Variants in individual genes were counted in the CSVD and gnomAD datasets and compared using Fisher’s exact test, and raw p values were extracted for a dominant and recessive model of inheritance. FDR calculations were then completed using the base statistics package in R, and samples with an FDR *p* value < 0.05 were considered as significant and investigate further for gene ontology, expression data and potential function as a cause of CADASIL-related CSVD.

## Results

### Over-representation tests

Merging all CADASIL-related CSVD WES files identified a total of 354,000 variants across the 50 samples. From these variants, filtering strategies identified *n* = 1928 variants across 1773 individual genes that were rare (MAF < 0.001) and predicted to have a functional effect. The over-representation test found that cell–cell adhesion processes (GO: 0098609) were the most significant PANTHER GO-Slim biological processes identified with an adjusted (FDR) *p* value = 1.52 × 10^–5^ (Table 1). There was also an under-representation of immune response and response to stimulus processes that were represented from this list.

**Table 1:** Results from the TRAPD Burden test identifying genes which there are an increased number of rare and functional mutations in compared to the gnomAD control dataset

Gene	Case count HET	Case total AC	Control count HET	Control total AC	*P*-value DOM	FDR DOM
TENM3	22	25	10,397	10,397	1.38E-11	3.63E-09
CNTN4	18	24	7943	7943	6.56E-10	8.63E-08
ARVCF	16	35	7463	7463	2.11E-09	1.85E-07
LRRC7	15	19	5896	5896	5.54E-09	3.22E-07
SCRIB	23	29	15,738	15,738	6.12E-09	3.22E-07
DSCAM	19	29	10,668	10,668	1.01E-08	4.43E-07
IGFN1	19	21	11,296	11,296	2.55E-08	9.58E-07
TENM2	19	27	12,757	12,757	3.05E-08	1.00E-06
PKP4	14	23	6099	6099	7.19E-08	2.10E-06
IGSF9	12	21	5399	5399	1.35E-07	3.56E-06
SDK2	21	34	15,515	15,515	1.50E-07	3.60E-06
BSG	13	13	5935	5935	3.97E-07	8.70E-06
CDH12	10	14	3385	3385	7.75E-07	1.57E-05
CDHR2	17	18	11,950	11,950	1.91E-06	3.58E-05
ROBO4	14	40	8300	8300	2.89E-06	5.07E-05
TENM4	16	19	11,316	11,316	4.61E-06	7.58E-05
DSP	16	22	11,613	11,613	6.43E-06	9.95E-05
DSCAML1	17	20	13,138	13,138	6.92E-06	0.000101133
FAT3	17	20	13,747	13,747	1.27E-05	0.000175345
CDHR3	10	12	4738	4738	1.49E-05	0.000196526
DLG2	12	13	7155	7155	1.84E-05	0.000230134
FAT1	19	32	17,582	17,582	2.22E-05	0.000265077
LGALS9C	6	11	3035	3035	2.52E-05	0.000288451
IL27RA	8	16	3235	3235	3.96E-05	0.000431162
CNTN2	11	18	6510	6510	4.10E-05	0.000431162
DLG4	9	10	4351	4351	4.95E-05	0.000500568
CELSR2	16	23	15,696	15,696	7.06E-05	0.000688141
MYPN	13	21	9718	9718	8.14E-05	0.000764551
DSG4	10	11	5991	5991	0.000107471	0.000974654
FBLIM1	6	7	1988	1988	0.000137799	0.001208036
SDK1	22	32	25,737	25,737	0.000146387	0.001241927
DLG1	9	19	5087	5087	0.000161883	0.001330475
DSC2	8	9	4548	4548	0.000405688	0.003154362
CNTN6	10	10	7070	7070	0.000407788	0.003154362
FAT2	15	17	15,114	15,114	0.000567347	0.004263207
CDH22	7	7	3768	3768	0.000702417	0.005131546
LRFN3	6	10	2735	2735	0.00074463	0.005286837
FAM49B	5	5	1808	1808	0.00076504	0.005286837
OBSL1	12	12	10,658	10,658	0.00078398	0.005286837
DSG1	8	11	5221	5221	0.000998318	0.006563942
IGSF21	6	6	4011	4011	0.005041989	0.032342515
PODXL	5	9	2880	2880	0.005693263	0.03565067
CDH10	5	5	2953	2953	0.006313734	0.038616556
DSC3	7	9	5689	5689	0.007026878	0.042001567
LIMS2	6	6	4350	4350	0.007409349	0.042362149
PODXL2	4	4	1934	1934	0.007369466	0.042362149
CDH6	5	5	3100	3100	0.007705581	0.043118467
CDH9	5	5	3163	3163	0.008363126	0.045151173
PTPRS	14	16	17,894	17,894	0.008412196	0.045151173

*HET* Heterozygous, *DOM* Autosomal dominant model, *FDR* False discovery rate

**Fig. 1:**
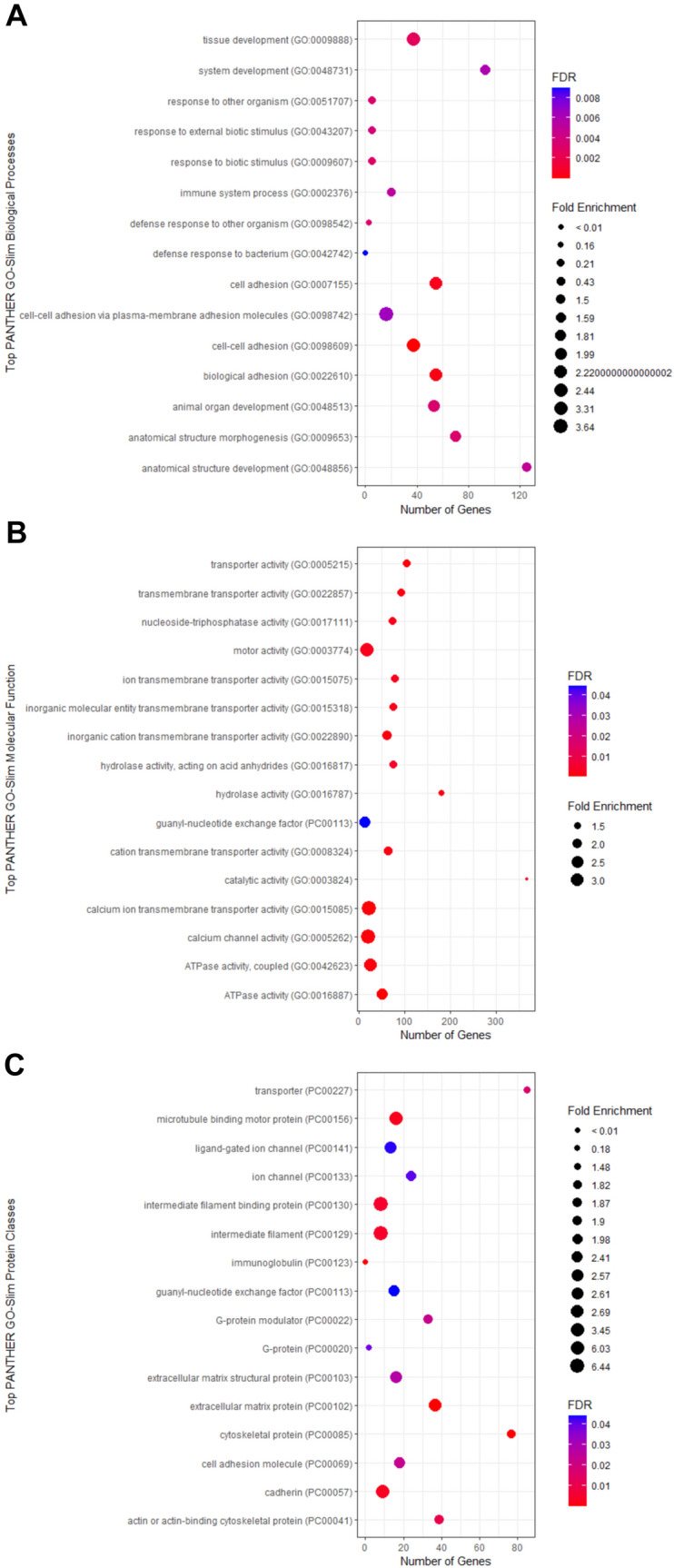
Results from the over-representation tests, investigating the top 15 hits for **A** PANTHER GO-slim biological processes, **B** PANTHER GO-slim molecular function, and **C** PANTHER GO-slim protein classes based on the PANTHER GO-slim overrepresentation test

Investigation of the PANTHER GO-Slim molecular function identified significant associations between ATPase activity (FDR corrected p value 7.52 × 10^–5^) and Ca^2+^ ion transmembrane transporter activity (FDR corrected p value 6.24 × 10^–4^) (Fig. 1). Ion channels and transport function genes comprised the most significant functions that were overrepresented in the cohort. Ion transmembrane genes and ATPase functions have been linked to numerous biological processes including cell signalling, adhesion and migration, and thus these results also reflect the PANTHER GO-slim biological processes (Fig. 1).

PANTHER Go-slim protein class output shows an increased number of genes involved in ECM proteins (PC00102) with a *p* value of 7.17 × 10^–5^ but interestingly a significantly decreased number of immunoglobulin genes (PC00123) *p* value 7.47 × 10^–5^ within our cohort that have rare and functional mutations. The number of ECM proteins as mutations in *COL4A1* and *COL4A2* has been identified to cause CSVD related to CADASIL. In conjunction with the biological processes and molecular function, there is also an overrepresentation of cell adhesion molecules (PC00069) *p* value 0.0232, Cadherin (PC00057) *p *value 0.00324 and microtubule-binding proteins (PC00156) *p* value 0.00283 which are involved in cell adhesion.

### TRAPD burden test

Based on these results, cell–cell adhesion was chosen as it was the top hit for the GO-PANTHER slim biological processes (FDR 1.52 × 10^–5^) and confirmation analysis using the different TOPPGene algorithm also found it highly significant (FDR 4.84 × 10^–5^). Cell–cell adhesion processes comprised 135 genes which was used in the TRAPD burden test from which 37 genes were identified in this cohort. Based on the results from the TRAPD burden test, 49 genes were identified as significant after multiple testing (FDR < 0.05) under the dominant inheritance model (Table 1) and 28 genes were identified as significant (FDR < 0.05) under the recessive inheritance model (Table 2) where 25 of these genes were identified as significant under the dominant and recessive models. Top hits under the TRAPD dominant model were identified as *TENM3* (FDR = 3.36 × 10^–9^)*, CNTN4* (FDR = 8.63 × 10^–8^),* ARVCF* (FDR = 1.85 × 10^–7^),* LRRC7* (FDR = 3.22 × 10^–7^) and *SCRIB* (FDR = 3.22 × 10^–7^)*.* For the recessive model, the top 5 hits included *ARVCF* (FDR = 8.39 × 10^–8^), *IGSF9* (FDR = 8.39 × 10^–8^), *PKP4* (FDR = 1.75 × 10^–7^), *ROBO4* (FDR = 1.75 × 10^–7^) and *LRFN3* (FDR = 6.80 × 10^–7^). From the top hits identified, only *ARVCF* and *PKP4* were in the initial candidate gene list. This is most likely due to the conservative nature of the TRAPD burden test which only accounted for MAF, SIFT and PolyPhen2 functional scores, whereas the initial list also incorporated further functional annotations from MutationTaster and PredictSNP2.

**Table 2:** Results from the TRAPD Burden test identifying genes which there are an increased number of rare and functional mutations in compared to the gnomAD control dataset based on the autosomal recessive model

Gene	Case count CH	Case count HOM	Case total AC	Control count HOM	CONTROL TOTAL AC	*P*-value REC	FDR REC
ARVCF	6	1	35	0	7463	6.26E-10	8.39E-08
IGSF9	5	1	21	0	5399	6.38E-10	8.39E-08
PKP4	6	0	23	0	6099	2.65E-09	1.75E-07
ROBO4	7	0	40	0	8300	2.66E-09	1.75E-07
LRFN3	4	0	10	0	2735	1.29E-08	6.80E-07
MYPN	7	0	21	0	9718	2.24E-08	8.29E-07
DLG1	5	0	19	0	5087	2.52E-08	8.29E-07
PODXL	4	0	9	0	2880	1.99E-08	8.29E-07
IL27RA	4	0	16	0	3235	4.80E-08	1.40E-06
CDH12	4	0	14	0	3385	6.84E-08	1.80E-06
DSC1	4	0	17	0	4159	3.44E-07	8.23E-06
SDK2	8	0	34	0	15,515	8.89E-07	1.95E-05
DSCAM	6	0	29	0	10,668	1.72E-06	3.48E-05
CNTN4	5	0	24	0	7943	1.90E-06	3.57E-05
LGALS9C	1	2	11	0	3035	4.07E-06	7.13E-05
CNTN2	4	0	18	0	6510	1.11E-05	0.00018198
TENM2	5	1	27	0	12,757	1.30E-05	0.000200786
FAT1	7	0	32	0	17,582	5.26E-05	0.000768088
DSP	5	0	22	0	11,613	7.02E-05	0.000971242
PALLD	3	0	9	0	5033	7.79E-05	0.001023798
CELSR2	5	1	23	0	15,696	0.000127292	0.001594175
LRRC7	3	0	19	0	5896	0.000195812	0.002340839
SCRIB	5	0	29	0	15,738	0.001119043	0.012796015
TNFRSF14	2	0	5	0	4229	0.001537552	0.016849005
DSG1	2	0	11	0	5221	0.00349771	0.036795905
TENM3	3	0	25	0	10,397	0.004962132	0.0466086
SDK1	7	0	32	0	25,737	0.004658739	0.0466086
DSC3	2	0	9	0	5689	0.0048463	0.0466086

*CH* Compound heterozygous, *HOM* Homozygous, *REC* Autosomal recessive, *FDR* False discovery rate

### Candidate cell–cell adhesion gene mutations

**Fig. 2:**
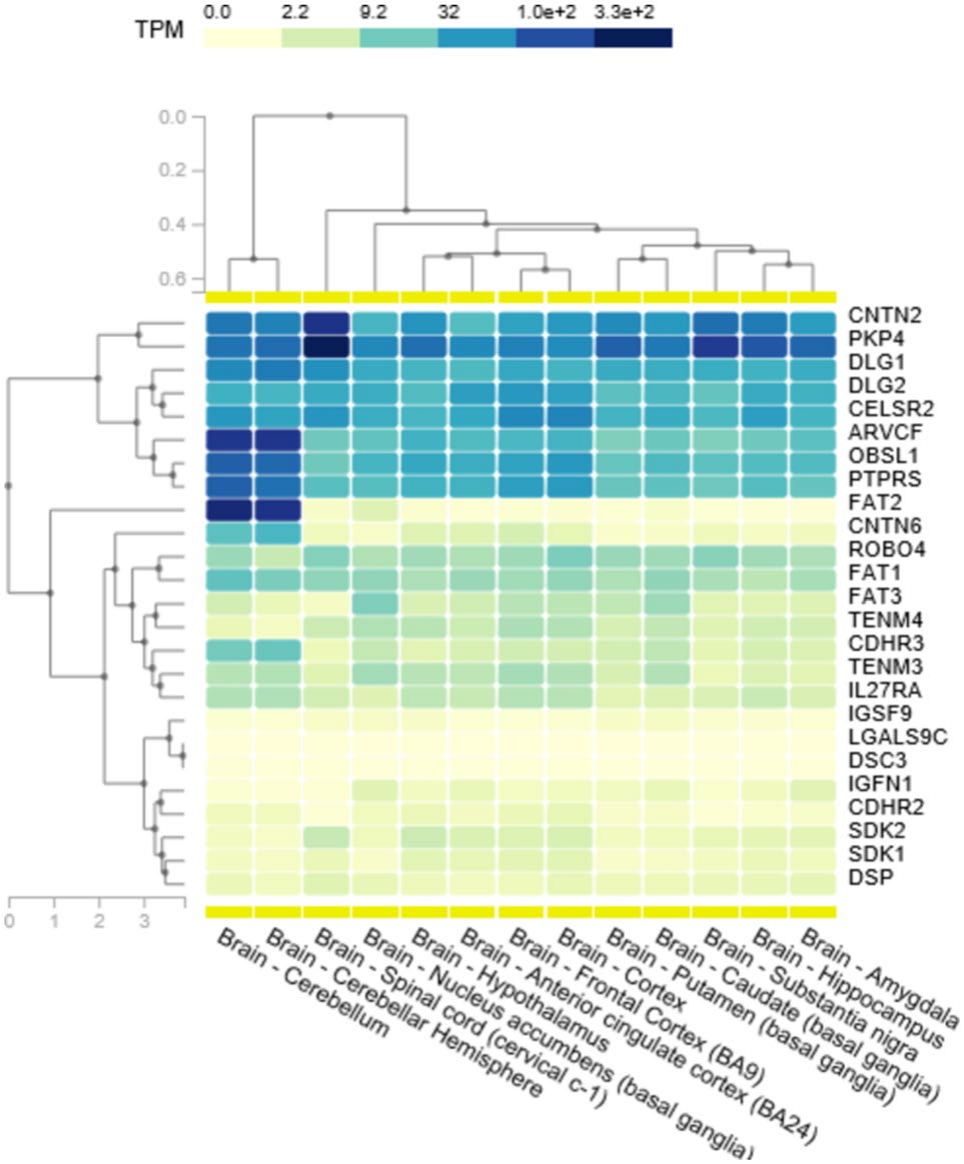
A gene expression map of brain tissues for the genes identified with candidate disease-causing mutations in the CADASIL-related CSVD cohort

The combined gene list of significant hits identified from both models included a total of 52 genes which was used for targeted WES analysis for novel causative mutations (Table 3). From this targeted analysis, there were 37 candidate mutations identified across 21 genes that met our criteria as potentially disease-causing (Table 3). Gene expression data obtained from GTEx shows *CNTN2*,* PKP4*,* DLG1*,* DLG2*,* CELSR2*,* ARVCF*,* OBSL1* and *PTPRS* as highly expressed across all brain tissues (Fig. 2). These were considered the more likely candidates based on expression profiles obtained. There were four mutations identified in *CNTN2*, which has also been associated with familial adult myoclonic epilepsy (a known uncommon symptom of CADASIL and other CSVD), indicating a potential overlap in the phenotypic spectrum of the disease. No other mutations in genes with high CNS expression had mutations identified in ClinVar.

**Table 3:**
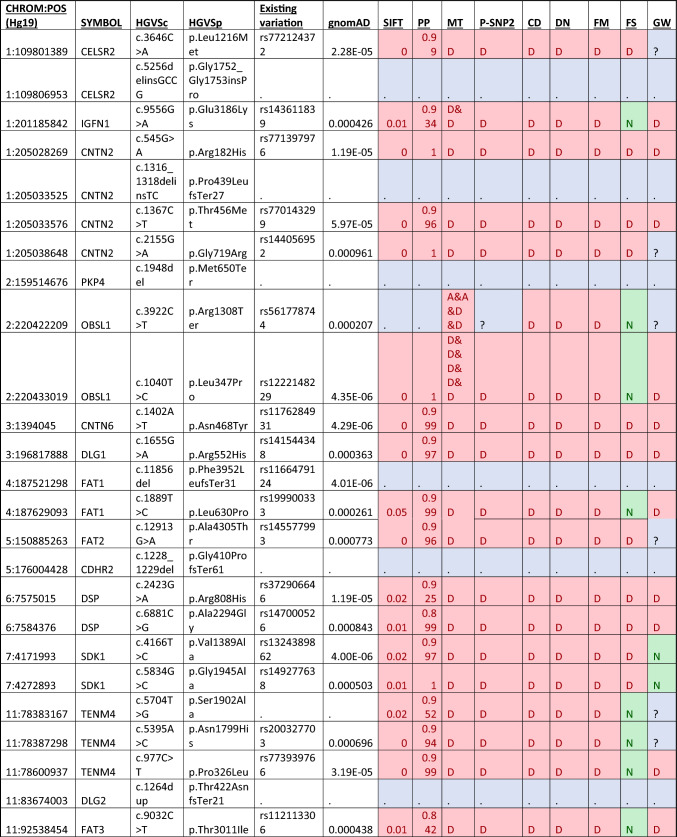

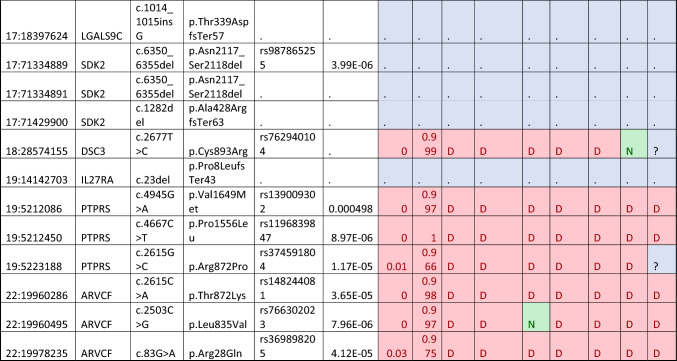
Candidate mutations identified in the cell–cell adhesion genes identified as significant after the TRAPD burden test

*PP* PolyPhen2, *MT* MutationTaster, *P-SNP2* PredictSNP2, *CD* CADD, *DN* DANN, *FM* FATHMM, *FS* FunSeq, *GW* GWAVA

## Discussion

Investigation of rare and functionally affected mutations in a cohort of 50 *NOTCH3* negative CSVD patients, showed an overrepresentation of genes involved in cell–cell adhesion. This indicates that there may be an increased role of cell–cell adhesion as part of the molecular mechanism of CADASIL-related disorders. This particularly matches previously literature which has shown that leaks in the tight junctions between the VSMCs are the cause of haemorrhagic strokes in some CADASIL patients (Dziewulska and Nycz 2016; Ling et al. 2019). Interestingly, results from the over-representation test matched a functional investigation of CADASIL-derived pluripotent stem cells (*NOTCH3* c.3226C > T p.R1076C), where cell–cell adhesion genes were shown to be over-expressed (Ling et al. 2019).

Cell–cell adhesion (GO: 0098609) has previously been investigated as a pathological feature of CADASIL and has been identified to show that VSMC adhesion to each other as well as the ECM is often impaired in some way (Tikka et al. 2012). VSMC adhesion complexes are altered in CADASIL patients, which cause an enlargement of the sub-endothelial spaces and a loss of intercellular connexions in CADASIL patients (Ruchoux et al. 1994, 2003). It has also been theorised that anoikis, a type of apoptotic cell death due to loss of appropriate cell adhesion to the ECM, may also play a role in CADASIL pathology (Dziewulska et al. 2017). Intercellular adhesion molecules have also been implicated to play a role in other cerebrovascular disorders, particularly adhesion markers related to inflammatory- and immune-mediated adhesions in large- and small-vessel diseases. These markers have also been implicated as a component of pathology in patients’ post-ischaemic stroke (Fassbender et al. 1999; Arba et al. 2019).

Investigation of the 138 cell–cell adhesion genes identified through from PANTHER through the TRAPD burden test identified 52 individual genes (49 under the dominant model and 28 under the recessive model) with significantly more mutations in this cohort compared to the gnomAD v2.1.1 exome controls. This large number of genes allowed further investigation of the rare and potentially functionally affected genes identified in our cohort as significant may be novel causes of CADASIL-related CSVD pathology. Through the combination of the rare variant-association strategy with targeted mutation analysis of the WES data, the number of mutations identified could be decreased from 1928 to 38. Further stratification based on mRNA expression data gives us some insight into potential biological relevance in brain tissue, which allowed us to focus on 17 variants across 8 genes (*CNTN2, PKP4, DLG1, DLG2, CELSR2, ARVCF, OBSL1* and *PTPRS*). All genes were also identified as associated using the TRAPD autosomal dominant model of inheritance which fits in with an initial diagnosis of CADASIL. Interestingly, only two genes (*OBSL1* and *PTPRS*) from the CNS highly expressed subset were not identified as significant via the recessive model as well.

*ARVCF*, encodes for Armadillo repeat protein deleted in velo-cardio-facial syndrome, is involved in cadherin-binding and protein–protein interactions at cellular junctions. *ARVCF* was identified as significant through three candidate heterozygous mutations detected were identified in the targeted analysis. An initial search of the literature failed to identify any interactions between *ARVCF* and *NOTCH3*, nor on any other well-characterised CSVD genes, such as *HTRA1*,* COL4A1* or *COL4A2. ARVCF* is highly expression in brain tissue; however, there is limited evidence to currently suggest a role that mutations within this gene plays in CADASIL or related CSVD pathology. This is in part due to knowledge related to the functional role of the protein being quite limited as well as a current lack of information relating to the gene’s role in neurological disorders.

*CNTN2* encodes for contactin-2, a glycoprotein which is highly expressed in various subsets of neuronal cells, predominantly on their axons (Dodd et al. 1988; Wolfer et al. 1994). This gene is predominantly known to cause the autosomal recessive familial adult-onset myoclonic epilepsy, 5 (FAME 5) (MIM#615,400), a disorder characterised by seizures, auditory or visual aura, depression and occasional cognitive deficits (Stogmann et al. 2013). Not only was *CNTN2* identified as significant through the TRAPD burden test, but there were four mutations identified which met the criteria as candidates for CSVD pathology. Three of the four mutations have previously been identified and been classified through ClinVar. This included the *CNTN2* c.545G > A rs771397976 and c.1367C > T rs770143299 which have been classified as VoUS (VCV000855428.2 and VCV000578301.4, respectively), and *CNTN2* c.5488G > A rs144056952 was likely benign (VCV000474475.4) for causing familial adult-onset myoclonic epilepsy (FAME 5). The other mutation was a novel *CNTN2* c.1316_1318delinsTC p.Pro439LeufsTer27) which would result in a truncated protein.

Interestingly, CNTN2 has been recognised as a ligand for APP that negatively modulates neurogenesis in a Notch-like fashion (Ma et al. 2008; Bizzoca et al. 2012). It was found that extracellular binding of CNTN2 to APP resulted in γ-secretase-dependant cleavage of the APP ICD. This interaction may be indicative of a pathogenic role in either CAA or Alzheimer’s disease pathogenesis. This link has also been investigated before where the SNPs rs10900451 and rs4950982 were associated with late onset of Alzheimer’s disease (LOAD) (Medway et al. 2010; Bamford et al. 2020). Furthermore, a link between *CNTN2* and more generalised neurodegeneration has been theorising based on the reduction of CNTN2 expression. It is thought that this reduced expression may predispose neurons to cell death, induced through the binding of TGFβ2 to APP (Tachi et al. 2010). Murine knockout of *Tag1* (murine *CNTN2* ortholog) found evidence of cognitive impairments based on the Morris water maze and novel object recognition tests, as well as reduced spontaneous motor activity, abnormal gait coordination and increased response latency to noxious stimulation (Savvaki et al. 2008). Moreover, Tag-1^−/−^ mice had shorter internodes in the cerebral and cerebellar white matter which were hypothesised to account for the behavioural deficits and hyperexcitability in these animals (Savvaki et al. 2008).

There were two mutations identified in *DLG1* (c.1655G > A p.Arg552His) and *DLG2* (c.1264dup p.Thr422AsnfsTer21) across two samples. These genes encode for Discs large MAGUK scaffold proteins 1 and 2 and may interact at post-synaptic sites to form multimeric scaffold for the clustering of receptors. To date, neither gene is known to be causative of any disease; however, *DLG1* has been associated with cleft-lip/palate and depression, and *DLG2* has been associated with schizophrenia and renal oncocytoma.

DLG1 expression changes have also previously been noted in a number of different pathologies including cancer, neurological and immunological disorders (Marziali et al. 2019). In neurological contexts, different mutations (including microdeletions, microduplications, methylation changes and single-nucleotide variants) that result in a DLG1 deficiency have been shown in schizophrenia, autism, Parkinson’s disease, epilepsy and cerebral palsy (Marziali et al. 2019). There has also been evidence in murine models that have shown that *Dlg1* knockout ameliorates depression-like behaviour. However, other studies found that heterozygous Dlg1 ± mice did not exhibit the behavioural deficits seen in mice harbouring the full 3q29 deletion (Rutkowski et al. 2021; Li et al. 2023). Despite these disease links, it remains unclear if this mutation is causative of, or contributing to, CSVD and further investigations of protein function would be required to validate this finding.

In contrast to *DLG1*, there is less evidence to suggest that *DLG2* is contributing to CSVD/neurodegenerative disease. The *DLG2* c.1264dup p.Thr422AsnfsTer21 mutation indicates a premature truncation of the protein and affects the PDZ-3 domain of the protein. More recently, variants and knockout studies of *DLG2* have been linked in to delayed puberty and autism spectrum disorders (Jee et al. 2020; Yoo et al. 2020). Despite the high expression in brain tissue, there does not seem to be evidence to suggest variants in the gene play a role in neurodegeneration, stroke or other CSVD symptoms.

There were two mutations in *CELSR2* (c.3646C > A p.Leu1216Met and c.5256delinsGCCG p.Gly1752_Gly1753insPro). Both mutations affect the extracellular domain of the CELSR2 protein, where only the p.Gly1752_Gly1753insPro is within a functional region, the Laminin G-like 1 domain. A role of CELSR2 has been linked to axonal guidance with implications of brain wiring in normal development and regeneration through many functional studies, including mouse knockout models, as well as playing a role in ependymal ciliogenesis (Tissir et al. 2010; Qu et al. 2014). Some studies have supported the hypothesis that *Celsr2* in adult mice helps maintain the integrity of the mature cortex, and that *Celsr2*-deficient mice have alteration in spinogenesis and reduced neuronal calcium activities (Li et al. 2022). This may indicate that changes to the protein structure could influence neuronal recovery, post-traumatic event. Also, *CELSR2* variants have been found to be associated with stroke and coronary artery disease through GWAS and meta-analyses; however, replication studies failed to confirm these findings (Dichgans et al. 2014; Zhou et al. 2015; He et al. 2016). Despite this, one study did show that the *CELRS2* may be associated with some serum lipid traits which may contribute to some form of vessel pathology (Zhou et al. 2015). Based on information from the mutations identified in *CELSR2* and investigations trying to link it to CSVD-based symptoms, it is unclear if mutations in this gene may be causative of CADASIL-related CSVD.

There were two mutations identified in *OBSL1* including the c.3922C > T p.Arg1308Ter (rs561778744) and c.1040 T > C p.Leu347Pro (rs1222148229). Whilst both mutations have previously been seen before, neither have been classified according to ClinVar. Interestingly, *OBSL1* has been identified as a critical regulator of Cullen-7, which is involved in the regulation of protein abundance (Litterman et al. 2011). This may indicate a role for both genes in CSVD-related pathology as excessive proteins have been seen in some monogenic forms of CSVD and CADASIL (Monet-Lepretre et al. 2013; Haffner 2019).

There were three mutations identified in *PTPRS* that were predicted as disease-causing and this included the c.4945G > A p.Val1649Met (rs139009302), c.4667C > T p.Pro1556Leu (rs1196839847) and c.2615G > C p.Arg872Pro (rs374591804). Whilst all mutations have been identified previously, there is no evidence to show they have been identified as causative of any disease by ClinVar. The *PTPRS* p.Pro1556Leu is one amino acid away from a binding site (position 1557) and is found in the Tyrosine protein phosphatase 1 domain of the protein. The p.Val1649Met mutation is not in a domain or repeat region of the gene, whereas the p.Arg872Pro change is within the Fibronectin type-III 6 section of the protein. It is unclear what effect these changes have on the protein and for CSVD-related symptoms as there is currently no evidence to suggest a contribution to pathology in humans. Mouse model *Ptprs* knockouts have shown severe neurological defects which overlap with some inherited CSVD conditions, including spastic movements, tremor, ataxic gait, abnormal limb flexion and defective proprioception (Wallace et al. 1999). Whilst this may indicate a role for *PTPRS* as a novel cause of CSVD, further work would be required to identify a causative link to this gene and CSVD phenotypes.

There were 20 genes which were identified as candidate causal mutations after the TRAPD burden test and subsequent targeted in silico prediction analysis. From these genes, 8 genes had high mRNA expression across the brain tissue. Investigations of *PKP4*,* DLG1*,* DLG2*,* PTPRS* and *OBSL1* found insufficient evidence to provide a direct link between these genes and neurodegenerative or stroke events. Other genes, such as *ARVCF*,* CELSR2* and *CNTN2*, had strong statistical significance, high gene expression in neural tissue and stronger potential links that suggest a role in CADASIL- or CSVD-related pathology. This highlights that there is some evidence to use a burden style statistical test, after the initial overrepresentation analysis is another way to filter for candidate mutations in WES approaches. However, the low number of case samples (*n* = 50) compared to the control dataset (*n* = 125,748) highlights that there was bias in the statistical calculations. It would be recommended for that this study be replicated in a larger CADASIL/CSVD cohort to validate any findings. Furthermore, the gene enrichment pathways that were identified only focussed on the top hit of cell–cell adhesion, as such, other pathways including as immune responses and response to stimulus (e.g. bacterial) were not investigated. Finally, the findings within this work should also be investigated further through segregation or functional studies using either cell lines (CRISPR techniques or patient derived lines) or animal studies (e.g. Zebrafish or *C. elegans*) to further elucidate a role for these genes in CADASIL-related disorders.

## Conclusion

Overall, this study identified cell–cell adhesion as the most significant overrepresented group in CADASIL-related disorders, according to the number of genes with rare and predicted disease-causing mutations. This system makes biological sense in CADASIL and related CSVD as adhesion of the VSMC and epithelial cells which comprise the vascular walls in the small vessels have often been identified to be disrupted in CADASIL-related CSVD. The higher number of mutations in cell–cell adhesion processes support to a role that a disruption caused by functional and rare gene mutations within this system may be a novel factor in CADASIL and CSVD pathology. Further statistical and candidate gene approaches also identified three genes (*CNTN2*,* CELRS2* and *ARVCF*) which may be novel causes of, or contributors to, CSVD pathology.

## Data Availability

All data relevant to this study are included within this manuscript. The raw case dataset has not been made readily available due to current ethical considerations related to patient privacy QUT HREC 1800000611. Access to this data may be made on request to the corresponding author, DProf Lyn Griffiths AM.
